# PlantDeepSEA, a deep learning-based web service to predict the regulatory effects of genomic variants in plants

**DOI:** 10.1093/nar/gkab383

**Published:** 2021-05-25

**Authors:** Hu Zhao, Zhuo Tu, Yinmeng Liu, Zhanxiang Zong, Jiacheng Li, Hao Liu, Feng Xiong, Jinling Zhan, Xuehai Hu, Weibo Xie

**Affiliations:** National Key Laboratory of Crop Genetic Improvement, Huazhong Agricultural University, Wuhan, China; National Key Laboratory of Crop Genetic Improvement, Huazhong Agricultural University, Wuhan, China; National Key Laboratory of Crop Genetic Improvement, Huazhong Agricultural University, Wuhan, China; National Key Laboratory of Crop Genetic Improvement, Huazhong Agricultural University, Wuhan, China; National Key Laboratory of Crop Genetic Improvement, Huazhong Agricultural University, Wuhan, China; National Key Laboratory of Crop Genetic Improvement, Huazhong Agricultural University, Wuhan, China; National Key Laboratory of Crop Genetic Improvement, Huazhong Agricultural University, Wuhan, China; National Key Laboratory of Crop Genetic Improvement, Huazhong Agricultural University, Wuhan, China; Hubei Key Laboratory of Agricultural Bioinformatics, College of Informatics, Huazhong Agricultural University, Wuhan, China; National Key Laboratory of Crop Genetic Improvement, Huazhong Agricultural University, Wuhan, China; Hubei Key Laboratory of Agricultural Bioinformatics, College of Informatics, Huazhong Agricultural University, Wuhan, China

## Abstract

Characterizing regulatory effects of genomic variants in plants remains a challenge. Although several tools based on deep-learning models and large-scale chromatin-profiling data have been available to predict regulatory elements and variant effects, no dedicated tools or web services have been reported in plants. Here, we present PlantDeepSEA as a deep learning-based web service to predict regulatory effects of genomic variants in multiple tissues of six plant species (including four crops). PlantDeepSEA provides two main functions. One is called Variant Effector, which aims to predict the effects of sequence variants on chromatin accessibility. Another is Sequence Profiler, a utility that performs ‘*in silico* saturated mutagenesis’ analysis to discover high-impact sites (e.g., *cis*-regulatory elements) within a sequence. When validated on independent test sets, the area under receiver operating characteristic curve of deep learning models in PlantDeepSEA ranges from 0.93 to 0.99. We demonstrate the usability of the web service with two examples. PlantDeepSEA could help to prioritize regulatory causal variants and might improve our understanding of their mechanisms of action in different tissues in plants. PlantDeepSEA is available at http://plantdeepsea.ncpgr.cn/.

## Graphical Abstract

**Graphical Abstract:**
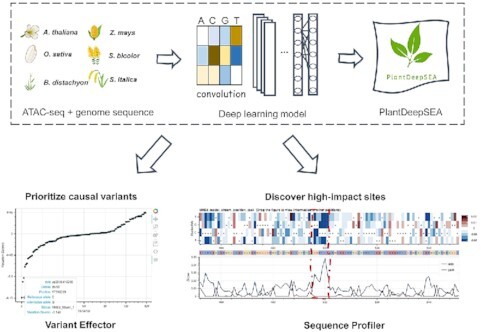
PlantDeepSEA integrates ATAC-seq data and genomic sequences based on a deep learning approach to predict the regulatory effects of genomic variants in multiple tissues of six plant species. It provides two main functions: "Variant Effector" to annotate the effects of genomic variants on chromatin accessibility, and "Sequence Profiler" to discover high-impact sites.

## INTRODUCTION

Quantitative trait locus (QTL) analysis and genome-wide association study (GWAS) have been widely used to dissect the genetic basis of complex traits in plants (1–4). However, since many neutral genomic variants are also significantly associated with traits in GWAS, it is difficult to determine causal variants based on association results alone. Furthermore, it is difficult to resolve the underlying mechanisms of variants, especially for non-coding variants (NCVs) (5). A recent review article summarized 364 QTLs cloned in six major crops and showed that in maize, 64% of the causal variants fall in non-coding regions (6), demonstrating the importance of the prioritization of NCVs and the annotation of *cis*-regulatory elements (CREs) in plant sciences.

With the development of high-throughput sequencing technologies, various assays have been developed to study epigenetic states at the genome-wide scale (7). And a large amount of high-throughput epigenetic data has been generated, offering the possibility of systematically modeling epigenetic states or regulations through machine learning approaches. With such models in place, we could predict epigenetic states from only genomic sequences. Then the regulatory effects of NCVs can be reasonably assessed by comparing the epigenetic state predictions obtained from sequences with the reference and the alternative genotypes, respectively. Furthermore, through an ‘*in silico* saturated mutagenesis’ approach, i.e. computationally mutating all nucleotides at each position, we can analyze the effects of each base substitution on epigenetic states, thereby identifying high-impact sites which are likely CREs (8).

Models based on deep neural networks (DNNs) have been proven to be powerful to automatically extract complex and relevant features from genomic sequences and to learn and predict epigenetic states accurately and efficiently (9). DeepSEA (deep learning-based sequence analyzer) (8), DeepBind (10), Basset (11) and Basset's successor, Basenji (12) are representative frameworks of DNNs. In comparison, DeepSEA has a simpler structure that allows training and annotating genomic segments and variants in a short time. In addition, a PyTorch-based deep learning library, Selene, makes it easy to build and train DNN models (13).

Compared to other epigenetic states, the prediction of chromatin accessibility or open chromatin exhibits higher accuracy in DNN models, with a median area under the curve of 0.923 in the human DeepSEA model (8). Meanwhile, open chromatin data are more easily obtained by techniques such as ATAC-seq (Assay of Transposase Accessible Chromatin sequencing) (7), and several datasets have been accumulated in plants (14–17). Based on open chromatin data it is easy to identify open chromatin regions (OCRs), which are considered to be the primary location of CREs (18). Causal variants in human GWAS are enriched in OCRs (19–22), and similar reports have been made in plants (23).

Although large-scale chromatin-profiling data have been available and several tools based on deep-learning models have achieved state-of-the-art performance in humans, no dedicated tools or web services have been reported in plants. In addition, for those who only want to prioritize causal variants or identify CREs in specific regions of a genome, building a deep learning model from scratch is very time-consuming and labor-intensive. Therefore, it is necessary to build an online web service to predict variant effects and CREs based on deep learning models in plants.

To this end, we present PlantDeepSEA (http://plantdeepsea.ncpgr.cn), an online web server for NCV prioritization and CRE identification in plants, built on high-quality chromatin accessibility data as well as the deep learning framework DeepSEA (8). The website offers two main functions. One is called Variant Effector, which aims to predict the effects of sequence variants on chromatin accessibility. Another is Sequence Profiler, a utility that performs ‘*in silico* saturated mutagenesis’ analysis to discover high-impact sites (e.g. CREs) within a sequence. The remainder of this paper presents the server in detail and demonstrates the usability of the web server with two examples.

## MATERIALS AND METHODS

### Chromatin accessibility data collection and quality control

We collected the ATAC-seq data of Arabidopsis (*Arabidopsis thaliana*) from the NCBI Sequence Read Archive (SRA) database with accessions SRP188687 (16), SRP111984 (17) and SRP113667 (24). The ATAC-seq data of *Brachypodium distachyon*, *Oryza sativa* cv. Minghui63 (*O. sativa-MH*), *O. sativa* cv. Zhenshan97 (*O. sativa-ZS*), *Setaria italica*, *Sorghum bicolor* and *Zea mays* were generated from our previously established protocol (25) and deposited in NCBI with SRA accession SRP308654.

The raw reads of ATAC-seq were first trimmed by Trimmomatic v.0.36 (26) with parameters of a maximum of two seed mismatches, a palindrome clip threshold of 30, and a simple clip threshold of 10, reads shorter than 30 bp were discarded. Then reads for each species were aligned to the reference genome (*A. thaliana*: TAIRv10.1, *B. distachyon*: Bd21-3 v1.1, *O. sativa-MH*: RS2, *O. sativa-ZS*: RS2, *S. italica*: v2.0, *S. bicolor*: v3.1.1 and *Z. mays*: AGPv4; the detailed information can be found at PlantDeepSEA website) using bwa v0.7.17 mem algorithm with parameter ‘-M -t 5 -k 32’ (27), respectively. Mapping reads with a mapping quality score below 30 and PCR duplicates, mitochondrial and chloroplast reads were filtered using SAMtools v.1.9 (28). To identified OCRs in each sample, narrow-peak calling settings were used in MACS2 v2.2.7.1 (29) with parameters ‘-g (1.2e8 for *A. thaliana*, 2.2e8 for *B. distachyon*, 3.0e8 for *O. sativa*, 3.4e8 for *S. italica*, 4.1e8 for *S. bicolor*, 9.5e8 for *Z. mays*) –nomodel –extsize 38 –shift -15 –keep-dup all -B –SPMR –call-summits’. Transcription start site (TSS) enrichment was calculated by counting fragments per base in the regions ±3000 bp surrounding TSSs of all annotated genes and dividing by the average fragment count of the 1,000 bp flanking ends.

### Model training in PlantDeepSEA

The deep learning framework DeepSEA implemented using Selene was used in this work (13). The architecture of the model is displayed in Supplementary Figure S1. The training, validation and test sets were generated with Selene IntervalsSampler function, with parameter ‘sample_negative: True, sequence_length: 1000, center_bin_to_predict: 200, feature_thresholds: 0.5, mode: train’. Each training sample is a 1000-bp sequence fetched from a reference genome, represented by a one-hot encoded matrix of length 1000 × 4, each of the four columns indicates a DNA nucleotide (‘A’, ‘G’, ‘C’ or ‘T’). For each ATAC-seq sample, the training sample is labeled as 1 (positive sample) if it overlaps with OCRs in this ATAC-seq sample by more than 50% of its length, otherwise it is labeled as 0 (negative sample). The model output is a vector of values from 0 to 1 represents the probability that the sequence belongs to OCRs in each sample. To ensure the independence of the training set from the validation set and the test set, data from 1 or 2 chromosomes were selected as the validation set or the test set, respectively (Supplementary Table S1), these chromosomes were excluded at the time of training. For each round of training, one-kilobase sequences (training data sets) were randomly selected within the specified sampling chromosomes. Subsequently, the validation set and the test set were selected from the chromosomes excluded from the training dataset, and the number of selected sequences was randomly selected in the ratio of ‘training set: validation set: test set’ = 8:1:1. The fraction of sequences labeled as OCR in the training and test sets ranged from 0.2–11.8% and 0.2–14.2%, respectively (Supplementary Table S2), which is consistent with the reported fraction of OCR on the genome in plants (16). Finally, we evaluated the performance of the model using the area under the receiver operating characteristic curve (AUROC) and precision-recall curve (AUPRC).

### Scoring the variants and *in silico* saturated mutagenesis analysis

*in silico* saturated mutagenesis scan of all possible nucleotide substitutions in an input sequence. Each substitution will generate a new sequence, and our model calculates the probability value that this sequence belongs to OCRs. We use the values of *P*_mut_ – *P*_ref_ to generate the *in silico* saturated mutagenesis heatmap, where *P*_ref_ represents the probability predicted for the original sequence and *P*_mut_ represents the probability predicted for the mutated sequence. We also compute the absolute values of *P_mut_* – *P_ref_*, and the log fold changes of odds, log(*P*_mut_/(1 – *P*_mut_)) – log(*P*_ref_/(1 – *P*_ref_)), which are stored in the ‘ism_abs_diffs.tsv’ and ‘ism_logits.tsv’ files. Users can download them from the results page.

To compute the chromatin effects of variants, for each variant, we obtain the 1,000-bp sequence centered on that variant from the reference genome which is used for the trained model. The probability value that the sequence carrying the reference or alternative allele at the variant position belongs to OCRs is then calculated separately. Similar to *in silico* saturated mutagenesis, we calculate the difference between the probabilities of the two genotypes, the absolute value of the difference, and the log fold changes of odds and store in files ‘diffs.tsv’, ‘abs_ diffs.tsv’ and ‘logits.tsv’, respectively. The scatterplot on the results page is generated using the difference value between the reference or alternative allele probabilities. All the scoring values can also be downloaded from the results page.

### Identification of regulatory motif occurrences

The position frequency matrix of regulatory motif was downloaded from PlantTFDB (30) and JASPAR 2020 (31). The motif occurrences were identified by the FIMO version 5.1 (32) with the *P*-value < 1e–4.

### Web server implementation

The web server is implemented using Django Web framework (https://djangoproject.com). Jobs are scheduled via Celery's asynchronous task queuing system (http://celeryproject.org/), with Redis (https://redis.io/) serving as a message broker, and executed on a Linux computer with 72 CPU cores and two GPU cores. All interactive charts were rendered with the bokeh (https://bokeh.org/) library. The tables were rendered with DataTables (https://datatables.net/).

## DESCRIPTION OF PLANTDEEPSEA

**Figure 1.**
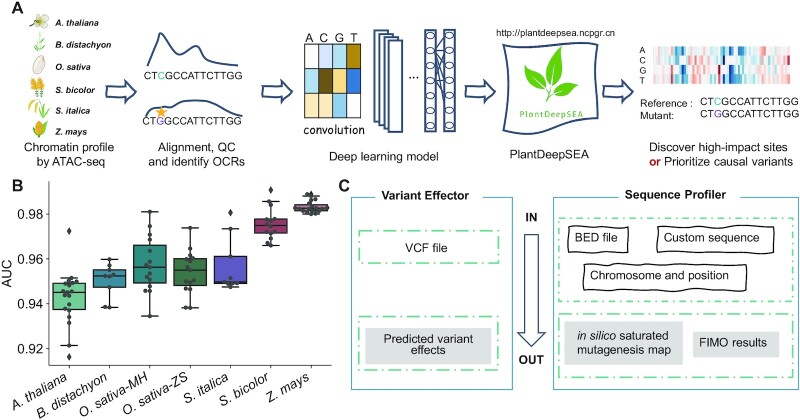
Overview of PlantDeepSEA. (**A**) Workflow of PlantDeepSEA. Firstly, we collected high-quality chromatin accessibility data from multiple representative tissues of six plant species. Secondly, we obtained credible open chromatin regions (OCRs) for each species through sequence alignment, quality control (QC), and OCR identification steps. Thirdly, we implemented a high-performance deep learning model, DeepSEA (8) using the Selene SDK (13), and used chromatin accessibility data to train the model. Fourthly, we built PlantDeepSEA (http://plantdeepsea.ncpgr.cn) based on tools such as Django and bokeh. PlantDeepSEA can be used to identify high-impact sites or prioritize causal variants. (**B**) Boxplot of area under curve (AUC) in each deep neural network model. Each point represents the corresponding AUC of each sample. (**C**) Two main functions in PlantDeepSEA. We designed two tools named ‘Variant Effector’ and ‘Sequence Profiler’, the accepted inputs and outputs are listed in the plot.

PlantDeepSEA provides an easy-to-use interface to prioritize NCVs and discover high-impact *cis*-regulatory sites within a sequence in plants (Figure 1A). Up to now, we have collected ATAC-seq data from multiple tissues of six representative plant species including Arabidopsis (*A. thaliana*), rice (*O. sativa*), maize (*Z. mays*), foxtail millet (*S. italica*), sorghum (*S. bicolor*) and *Brachypodium distachyon* (*B. distachyon*), and obtained OCRs in different tissues of these species (Table 1, Supplementary Table S2). We then implemented a published deep learning framework, DeepSEA (8) using the Selene library (13) and used OCRs to label hundreds of thousands of 1000-bp sequences and train the model for each species. We eventually obtained seven trained models, two models for rice and one model for each of the other species. The model output is a vector of values from 0 to 1 represents the probability that the sequence belongs to OCRs in each sample. When validated on independent test sets, AUROC of each model ranged between 0.93 and 0.99 (Figure 1B, Supplementary Figure S2 and Supplementary Table S2) and AUPRC ranged between 0.25 and 0.77 (Supplementary Figure S3 and Supplementary Table S2), which is similar to that reported in human models (8,13). Compared with the fraction of positive samples in test sets (range 0.2–14.2%), the values of AUROC and AUPRC demonstrate the usability of the models used in PlantDeepSEA.

**Table 1.** Summary statistics of ATAC-seq data used in PlantDeepSEA

Species	Tissue number	Sample number	Total Q30 read number^a^	Mean TSS enrichment^b^	Mean OCR number^c^
***A. thaliana***	6	14	458 734 749	12.1	25 947
***B*.*distachyon***	5	9	187 359 453	11.5	44 370
***O. sativa-MH***	6	15	625 034 398	12.0	75 670
***O. sativa-ZS***	6	15	521 213 434	13.9	72 567
***S*.*italica***	5	9	624 666 196	7.2	72 230
***S. bicolor***	7	14	818 482 967	9.9	82 166
***Z. mays***	8	19	856 301 588	11.0	74 257

^a^The total number of reads per sample aligned to the reference genome (mapping quality >30).

^b^Mean TSS enrichment score for each sample.

^c^Mean of the number of OCRs identified by MACS2 in each sample.

To evaluate the ability of PlantDeepSEA to predict the tissue specificity of OCRs, we calculated the Shannon entropy using the prediction scores for each sequence labeled as OCR in at least one sample in the test set. A small Shannon entropy indicates tissue specificity (33). The results show that the sequences labeled as OCR in fewer samples have smaller Shannon entropy (Supplementary Figure S4), indicating that PlantDeepSEA has the ability to predict the tissue specificity of OCRs at least to some extent.

Based on these trained models, we have designed a series of online tools to help users quickly obtain predicted results for genomic variants or interested regions. After selecting a model listed on the home page, the user can use ‘Variant Effector’ to predict the regulatory effects of variants in different tissues, or use ‘Sequence Profiler’ to judge whether the submitted sequence belongs to OCRs and to identify putative CREs (Figure 1C). After submitting the task, the user will be given a job ID and will be redirected to a page that will automatically refresh. The results are displayed on this page when the task is completed, and the user can also use the job ID to query the results within a week.

### Variant effector

Variant Effector is a tool designed for predicting the effects of sequence variants on chromatin accessibility. The accepted input is a VCF file containing information on the sequence variants. The results contain information on the effects of variants on chromatin accessibility in each tissue. Each variant has an effect score for each tissue, calculated as the predicted probability that the alternative allele belongs to OCRs in this tissue minus the predicted probability that the reference allele belongs to OCRs in this tissue. In the first part of the result page, variants are plotted and ranked by the effect scores. The second part of the result page is a table containing the effect scores of variants, genotypes, and tissue information (Figure 1C). The user might prioritize the variants by referring to the ranking of their effect scores. All results can be downloaded as figures or tsv-files.

### Sequence profiler

Sequence Profiler is a utility that performs ‘*in silico* saturated mutagenesis’ analysis for discovering high-impact sites within a sequence. Specifically, it performs computational mutation for every base of the input sequence and predicts the effect of every mutation on chromatin accessibility. The accepted inputs are a chromosome and a position, a BED file containing multiple coordinates of genomic regions or a custom sequence. The way of submitting custom sequences to Sequence Profiler can be used to predict the effect of haplotypes, i.e., one can evaluate the effect of different combinations of variants and the effect of variants in different sequence contexts. Details of the calculations and presentation of results are given in Materials and Methods and the figure legend (Figure 1C).

## CASE STUDIES

### Prioritizing non-coding causal polymorphisms in the rice gene *DEP1*

**Figure 2.**
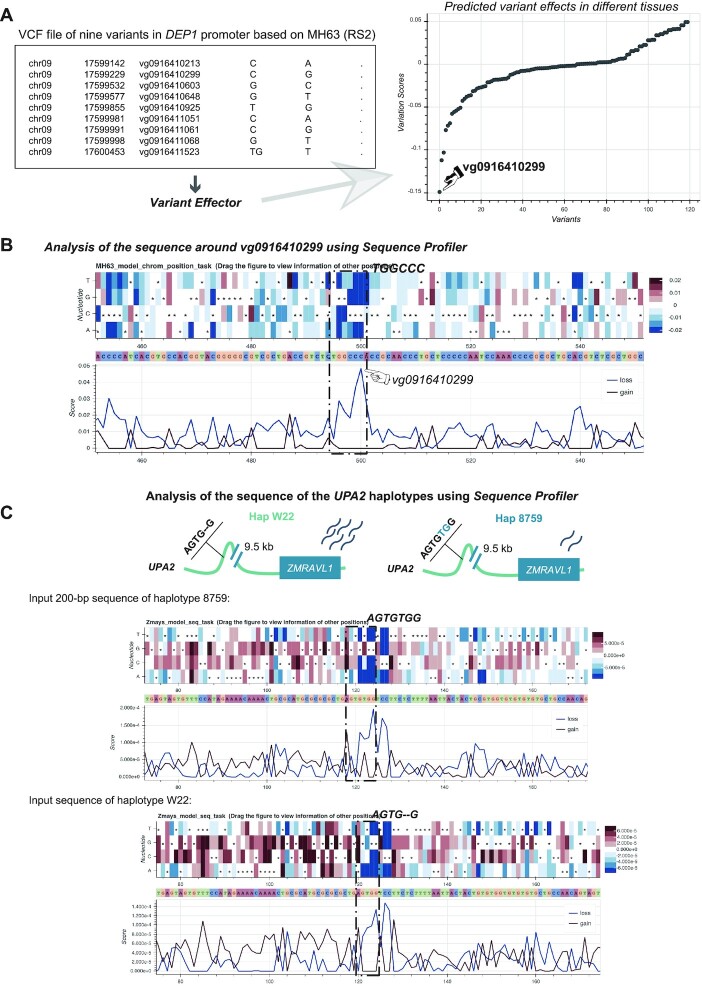
Two case studies. (**A**) Prioritization of causal variants in *DEP1* promoter region. We made the VCF file of nine variants in *DEP1* promoter region and used the tool ‘Variant Effector’ to prioritize these variants. The result showed that vg0916410299 was ranked as the most likely causal variant among the provided variants. (**B**) Analysis of high impact sites around the SNP vg0916410299. We used the tool ‘Sequence Profiler’ by entering the chromosome and the position of vg0916410299. The *in silico* saturated mutagenesis map showed sequence TGGCCC (overlapped with vg0916410299) might be a *cis*-regulatory element. (**C**) Analysis of high impact sites for different haplotypes of QTL *UPA2* using the tool ‘Sequence Profiler’. The *in silico* saturated mutagenesis map of CIMMYT 8759 haplotype (upper) and W22 haplotype (under) showed the sequence AGTGTG might be a *cis*-regulatory element, which is consistent with the results of Tian *et al.* (39). The loss score refers to the maximum decrease in probability that an allele belongs to open chromatin compared to the reference nucleotide in all mutations at each site. And the gain score refers to the maximum increase.

*DEP1* is a well-studied gene in rice, which regulates leaf and panicle morphology and has been widely used in rice breeding for high yield (34). A recent study showed that nine NCVs in the *DEP1* promoter region (2.0 kb upstream of the ATG) can regulate the gene expression and leaf-trait variation (35). We mapped these nine variants to RiceVarMap database (36) and constructed the VCF file based on the reference genome Minghui 63 (RS2). We first selected the model ‘Minghui 63’ listed on the home page and then selected the corresponding reference genome ‘Minghui 63 (RS2)’ listed in the panel ‘Variant Effector’. Then we uploaded the VCF file to Variant Effector to predict the effect of the variants. From the results, we found one SNP, named vg0916410299 in RiceVarMap, had the greatest effect score compared to the other variants (Figure 2A). We next inputted the genomic coordinates of this SNP into the panel ‘Sequence Profiler’. In the result page, the *in silico* saturated mutagenesis map showed that the sequence TGGCCC, which overlaps with vg0916410299, has the extreme effect scores (Figure 2B). FIMO (32) results also indicate that this sequence overlaps with a binding motif of the TCP transcription factors (37). We also noticed that another study reported the vg0916410299 as the only variant in the *DEP1* promoter associated with panicle traits (38), which is consistent with the prediction results.

### Discovering high-impact sites within the maize QTL *UPA2*

Maize leaf angle is an important factor affecting maize plant density and yield. Tian et al delimited a QTL *UPA2* to a 240-bp non-coding region using a BC_2_S_3_ population constructed by crossing a teosinte line CIMMYT 8759 with a maize inbred line W22 (39). They finally confirmed that a 2-bp deletion in the C_2_C_2_ motif (AGTGTG) is the functional variant regulating a gene *ZmRAVL1* located 9.5 kb downstream. We used the tool Sequence Profiler to analyze the two haplotypes of the 200-bp region around the 2-bp deletion. The *in silico* saturated mutagenesis map in the flag leaf (rep1) shows the haplotype of CIMMYT 8759 (with AGTGTG) has intensive high effect scores in the C_2_C_2_ motif region compared to the haplotype of W22 (with AGTG–) (Figure 2C).

These case studies demonstrate that PlantDeepSEA could help to identify causal NCVs and functional CREs.

## DISCUSSION

In this work, we constructed PlantDeepSEA, a deep learning-based web service to predict regulatory effects of genomic variants in plants for users with or without DNNs expertise. In each step of the analysis process, we used various rigorous criteria to evaluate the quality of the data and DNN models in PlantDeepSEA, and we designed several useful and user-friendly tools and have shown how to use the website by case studies. For reasons of data availability and uniformity, we currently support only six representative plant species and construct the models using only chromatin accessibility data. More species and more chromatin features will be integrated in future updates. Moreover, due to the limitation of computational resources, we have limited the length of the analyzed sequences and the number of analyzed intervals, which may be gradually solved in the subsequent updates. We also note that some recently published DNN models such as Basenji (12) may yield more accurate prediction results, and deepLIFT (40) can detect high-impact sites more efficiently by the backpropagation-based approach. We will integrate more DNN methods and applications in the future to comprehensively evaluate CREs as well as NVC effects in sequences. We believe that PlantDeepSEA will greatly facilitate the prioritization of regulatory causal variants and help to improve our understanding of their mechanisms of action in different tissues in plants.

## DATA AVAILABILITY

PlantDeepSEA (http://plantdeepsea.ncpgr.cn/) is freely available to all users. The sequences of reference genomes used in PlantDeepSEA, the OCR lists identified from ATAC-seq data, the deep learning models and the configure files used for model training can be accessed at https://plantdeepsea-toturial2.readthedocs.io/en/latest/08-Statistics.html.

## Supplementary Material

gkab383_Supplemental_Files
