# Supplementary material for: PlantDeepSEA, a deep learning-based web service to predict the regulatory effects of genomic variants in plants
Source: Nucleic Acids Res. 2021 May 25;49(W1):W523–9. doi: 10.1093/nar/gkab383 (PMC8262748; doi:10.1093/nar/gkab383)
Supplement: gkab383_Supplemental_Files [file gkab383_supplemental_files.zip › Supplementary Figures.pdf]

Model Architecture:

1. Convolution layer (320 kernels. Window size: 8. Step size: 1.)
2. Convolution layer (320 kernels. Window size: 8. Step size: 1.)
3. Pooling layer (Window size: 4. Step size: 4.)
4. Convolution layer (480 kernels. Window size: 8. Step size: 1.)
5. Convolution layer (480 kernels. Window size: 8. Step size: 1.)
6. Pooling layer (Window size: 4. Step size: 4.)
7. Convolution layer (960 kernels. Window size: 8. Step size: 1.)
8. Convolution layer (960 kernels. Window size: 8. Step size: 1.)
9. Fully connected layer (target nums)
10. Sigmoid output layer

Regularization Parameters:

Dropout proportion (proportion of outputs randomly set to 0):

- Layer 6: 20%
- Layer 8: 20%
- All other layers: 0%

Batch normalization applied after layers 3, 6, and 8 and before dropout.

Model architecture use the binary cross-entropy loss function and stochastic gradient descent optimizer (momentum, 0.9; weight decay, 10−6).

Supplement Figure S1. PlantDeepSEA model Architecture.

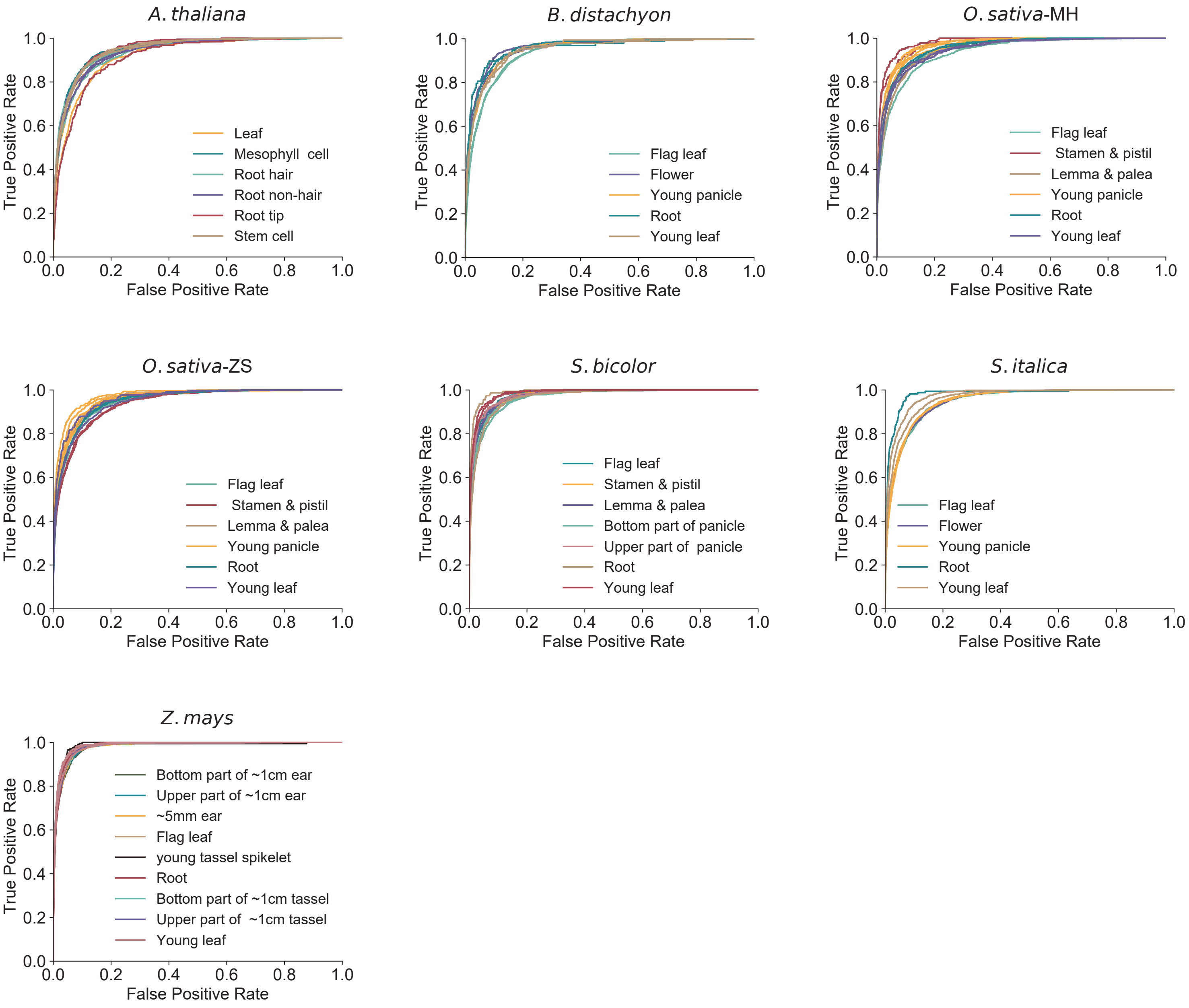

Supplement Figure S2. Receiver operating characteristic curves of each deep neural network model.

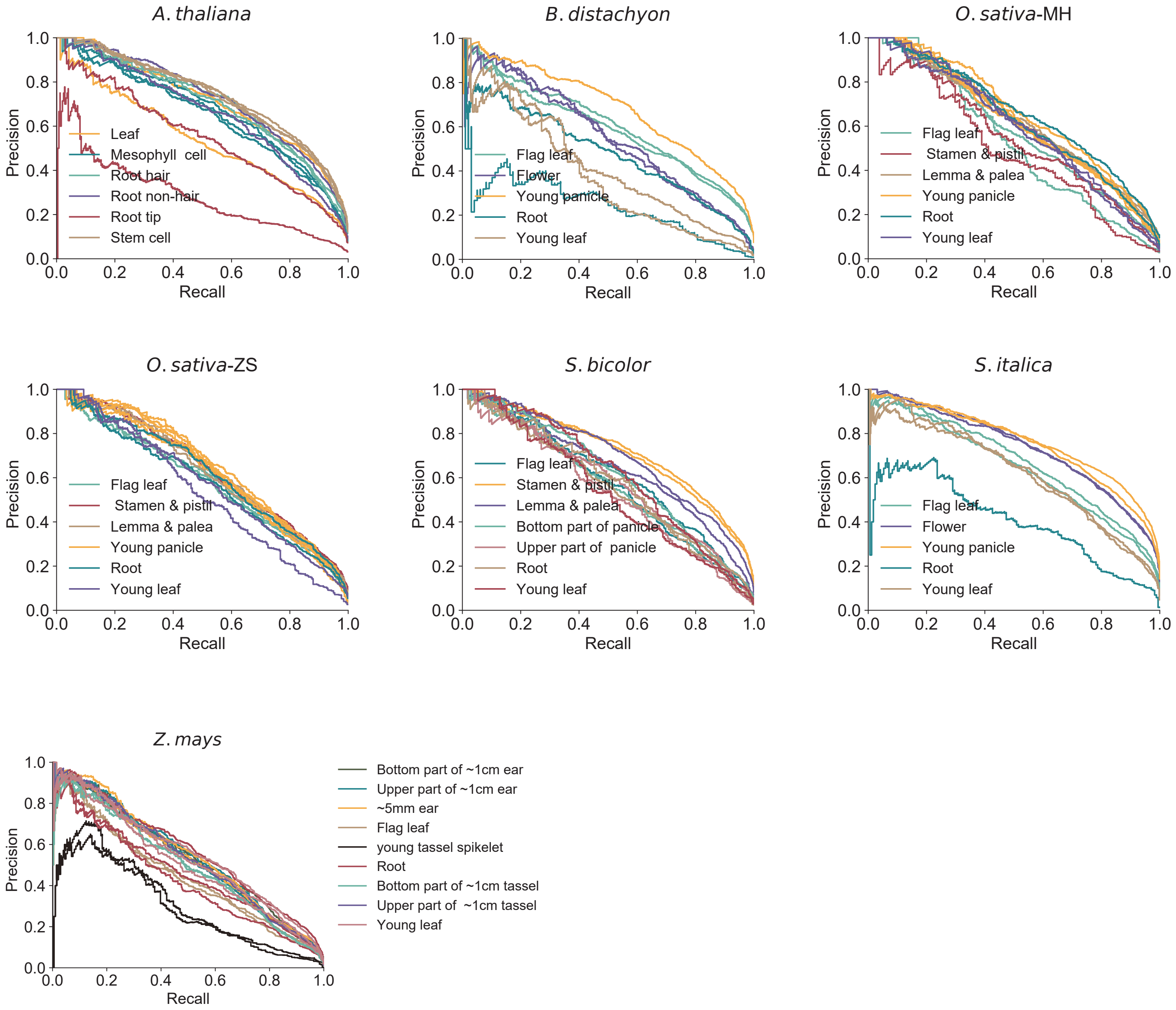

Supplement Figure S3. Precision-recall curves of each deep neural network model.

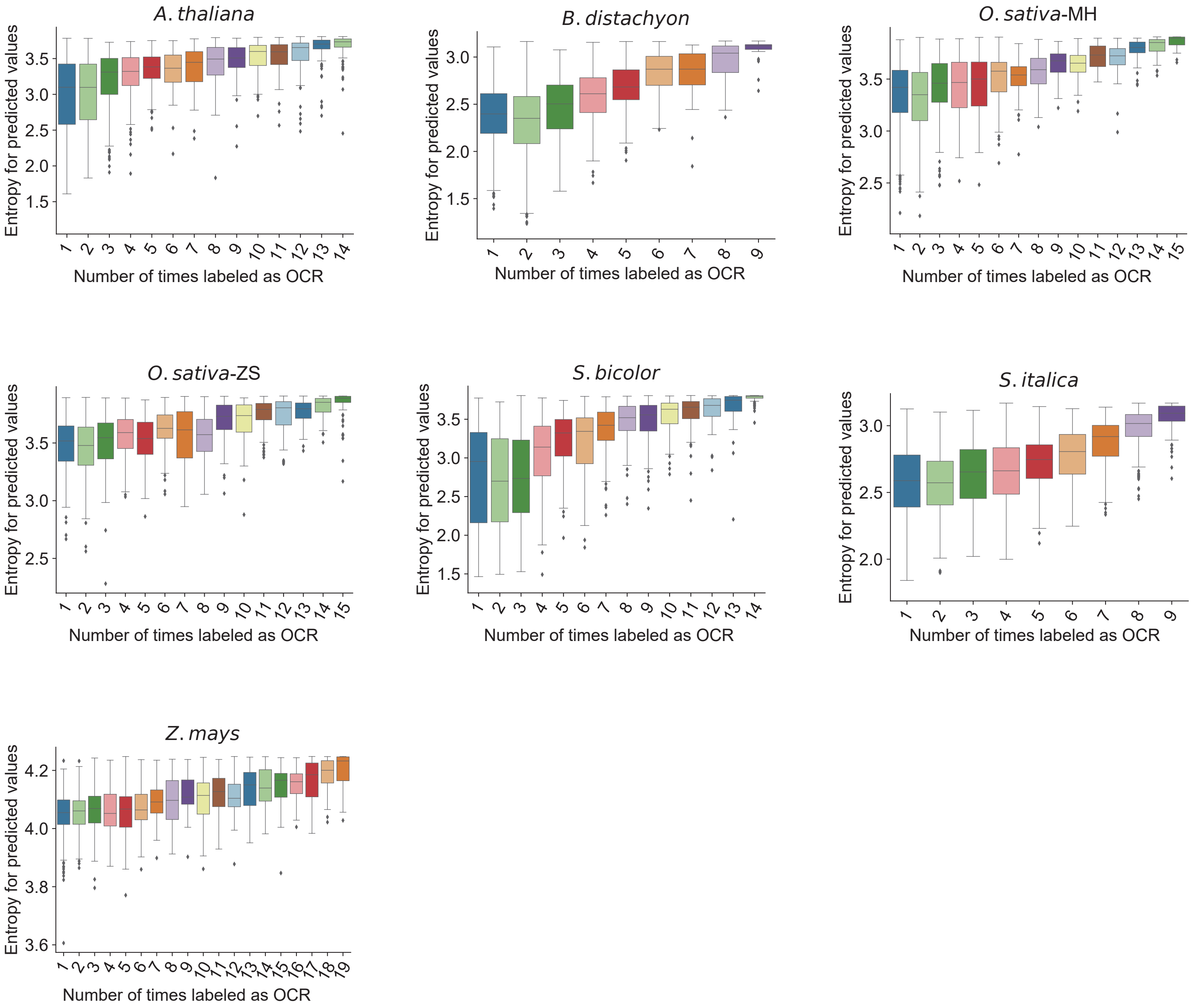

Supplement Figure S4. Boxplots of the number of times a sequence is labeled as OCR in different samples versus the Shannon entropy of the predicted values of that sequence. Sequences labeled as OCR in at least one sample in the test set were used.
